# Bardet-Biedl syndrome proteins control cilia length through regulation of actin polymerisation

**DOI:** 10.1186/2046-2530-1-S1-P88

**Published:** 2012-11-16

**Authors:** V Hernandez, P Pravincumar, A Diaz-Font, H May-Simera, D Jenkins, M Knight, PL Beales

**Affiliations:** 1 Molecular Medicine Unit, UCL Institute of Child Health London, UK; 2 School of Engineering and Materials Science, Queen Mary University of London, London, UK; 3 National Institute of Deafness and Communication Disorders, NIH, Bethesda, MD, USA

## 

Primary cilia are cellular appendages important for signal transduction and sensing the environment. Bardet-Biedl syndrome proteins form a complex that is important for several cytoskeleton-related processes such as ciliogenesis, cell migration and division. However, the mechanism by which BBS proteins may regulate the cytoskeleton remain unclear. We discovered that *Bbs4* and *Bbs6* deficient renal medullary cells display a characteristic behaviour comprising poor migration, adhesion and division with an inability to form lamellipodial and filopodial extensions. Moreover, few cells were ciliated (48% ± 6 for WT cells vs 23% ± 7 for *Bbs4* null cells) and bear shorter cilia (2.55 μm ± 0.41 for WT cells vs 2.16 μm ± 0.23 for *Bbs4* null cells). Whilst the microtubular cytoskeleton and cortical actin was intact, the actin cytoskeleton was severely disrupted, forming abnormal apical stress fiber aggregates. Furthermore, we observed over-abundant focal adhesions in *Bbs4*, *Bbs6* and *Bbs8*-deficient cells. In view of these findings and the role of RhoA in regulation of actin filament polymerisation, we showed that RhoA-GTP (activated form) levels were highly upregulated in the absence of Bbs proteins. Upon treatment of *Bbs4*-deficient cells with a RhoA inhibitor, Y27632, we were able to restore cilia length and number as well as the integrity of the actin cytoskeleton. Together these findings indicate that Bbs proteins play a central role in the regulation of the actin cytoskeleton and control cilia length through alteration of RhoA levels.

